# Dramatic decreases of all haemorrhagic coagulation factors by acquired inhibitors after extended left lobectomy

**DOI:** 10.1016/j.ijscr.2019.01.020

**Published:** 2019-01-30

**Authors:** Yuhki Sakuraoka, Takashi Suzuki, Takatsugu Mtsumoto, Genki Tanaka, Takayuki Shimizu, Takayuki Shiraki, Park Kyongha, Shozo Mori, Yukihiro Iso, Masato Kato, Taku Aoki, Keiichi Kubota

**Affiliations:** Second Department of Surgery, Dokkyo Medical University, 880 Kitakobayashi, Mibu, Tochigi 321-0293, Japan

**Keywords:** CT, computed tomography, PT%, prothrombin percentage, PT-INR, prothrombin time-international normalized ratio, APTT, activated partial thromboplastin time, ALP, alkaline phosphatase, CEA, carcinoembryonic antigen, CA19-9, cancer antigen 19-9, ERCP, endoscopic retrograde cholangiopancreatography, UICC, union for international cancer control, POD, post operative days, PSL, alpha-fetoprotein, PTCD, percutaneous trans-hepatic cholangiodrainage, FFP, fresh frozen plasma, Acquired inhibitors of coagulation factors, Liver resection

## Abstract

**Introduction:**

Acquired inhibition of coagulation factors is a rare disease, and the diagnosis is often difficult and delayed. We experienced a deficiency in all coagulation factors after hepatobiliary surgery.

**Case Presentation:**

Extended left liver resection was undertaken and hepaticojejunostomy was performed in a 70-year-old man. He had suffered from a high fever caused by cholangitis for 35 days. The major cause was a narrowing of the hepaticojejunostomy, and reconstruction was carried out. Twenty-four days later, there was a sudden massive bleed from his nose and the surgical site. Steroid pulse therapy was used as a treatment because cross mixing and some blood tests revealed the patient was experiencing an inhibition of all coagulation factors, and consequently the levels of coagulation factors dramatically recovered.

**Discussion:**

We considered malignancy and surgical damages to be the underlying cause. The reported treatment and examination will help clinicians explore additional reasons for massive bleeding after a severe physical injury.

**Conclusion:**

We have described the first case of acquired inhibition of all coagulation factors associated with extended left lobectomy.

## Highlights

• Required inhibitors of all coagulation factors.
• After liver resection.
• Severe bleeding.
• Benefits of using steroid.

## 1 Introduction

Acquired inhibition of blood coagulation factor is a rare disease, and the literature has revealed that the causes are a deficiency in coagulation factor V, VIII or XIII (13) [1]. With regard to factor V, there have been almost 100 reported cases around the world since the first description of this disease in 1955 [2]. A few reports have described some specific types of surgery that lead to the production of these inhibitors among elderly patients. More specifically, Chouhan showed that fibrin glue with a small amount of coagulation factor V led to the production of the antibody [3]. Meanwhile, for factor VIII, Matzinger explored a specific danger signal that could possibly be the vital factor of acquired haemophilia because of the emission of this signal from damaged cells, which was caused by infection and the erosion of tissue [4]. Moreover, some previous literature has reported massive bleeding associated with acquired inhibition of factor XIII (13), although the mechanism is still unknown [5].

We experienced massive bleeding after the operation for hilar cholangiocarcinoma, and this bleeding was associated with acquired inhibition of coagulation. We were able to prevent irreversible damage by using steroids. We consider our present case as extremely rare because the patient expressed inhibitors of all coagulation factors and there have been no previous reports of this nature worldwide. The work has been reported in line with the SCARE criteria [6]. We hope our case report will help clinicians to consider an uncommon cause of bleeding after surgery.

## 2 Case presentation

**Fig. 1:**
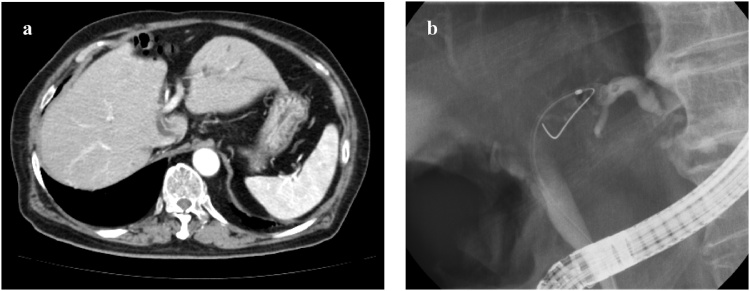
Abbreviations; ERCP: Endoscopic retrograde cholangiopancreatography, CT: computed tomography (a) CT imaging demonstrated dilatation of peripheral side of the left bile ducts. (b) ERCP revealed left-sided bile duct was narrow and the peripheral bile ducts were dilatation. Brushing cytology was performed at the narrow portion, and there was not malignancy in the pathological results.

A 70-year-old man visited a local clinic with abdominal pain. Blood biochemistry showed marginally high levels on a liver function test, and computed tomography (CT) imaging revealed dilatation of the peripheral left bile ducts (Fig. 1a). Subsequently, he was referred to our department. On admission, the patient’s body temperature was 35.9 °C, and the patient had no abnormal findings in the neck or thoraco-abdominal region. Blood tests showed no abnormalities, including prothrombin time percentage (PT%) and activated partial thromboplastin time (APTT), but blood biochemistry revealed that there was a slight increase in the level of alkaline phosphatase (ALP): 440 U/L. The total bilirubin level was 0.6 mg/dL. Examination of tumour markers revealed a carcinoembryonic antigen (CEA) level of 0.9 ng/mL and a level of cancer antigen 19-9 (CA19-9) was within normal range (6 U/mL) (Table 1).

**Table 1:** Laboratory data.

Variable	Pre-operative data	Post-operative data	Normal range
GOT (U/L)	30	92	13–30
GPT (U/L)	29	28	7–30
γ-GTP (mU/mL)	**200**	521	9k32
ALP (U/L)	**440**	**2815**	106k322
T-Bil (mg/dL)	0.6	**1.7**	0.4k1.5
CEA (ng/mL)	0.9	–	<5.0
CA19-9 (U/mL)	6	–	<37
WBC (×10^9^/l)	6.1	**11.40**	3.3–6.6
RBC (×10^12^/l)	4.4	**3.24**	4.35–5.55
Hb (g/dl)	13.9	**9.7**	13.7–16.8
Ht (%)	45.8	**29.7**	40.1–50.1
Plt (×10^4^/μl)	18.4	**7.8**	15.8k34.8
PT (%)	82	**7**	>70
APTT (sec)	36.1	**>180**	<60
P-FDP (μg/ml)	–	**11.1**	2.0～8.0
D dimer (ng/ml)	–	**7.9**	<150
AT3 (%)	–	**60**	80–120

Abbreviations: GOT: glutamate oxaloacetate transaminase, GPT: glutamate pyruvate transaminase, γ-GTP: γ-glutamyl transpeptidase, ALP: alkaline phosphatase, T-Bil: total bilirubin, CEA: carcinoembryonic antigen, CA19-9: cancer antigen 19-9, WBC : white blood cell count, RBC: red blood cell count, Hb: haemoglobin, Ht: haematocrit, Plt platelet, PT: prothrombin percentage, APTT: activated partial thromboplastin time P-FDP: plasma-fibrinogen fibrin degradation product, AT3: antithrombin III.

**Fig. 2:**
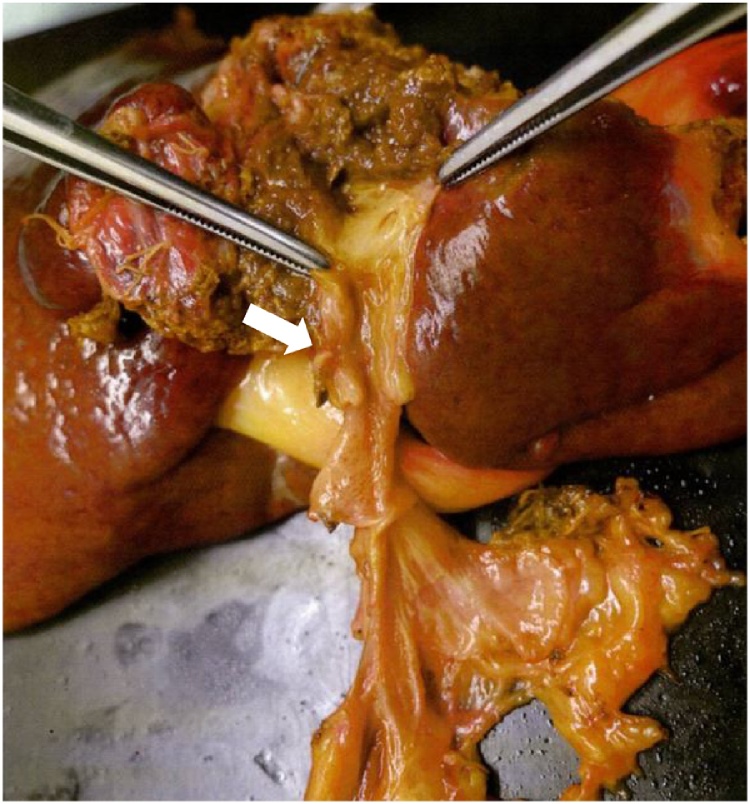
Extended left lobectomy was performed and hepaticojejunostomy was undertaken as the reconstruction. Histopathological findings revealed uncertain the tumour diagnosed with biliary intraepithelial neoplasia was located in hilar left part of bile duct as the white arrow shown.

Endoscopic retrograde cholangiopancreatography (ERCP) revealed disruption of contrast medium flow at the hilar part, and enhanced CT showed there was dilatation of left bile duct (Fig. 1). Although brush cytology at the site of the distal bile duct stricture was not scored as class V (adenocarcinoma), we diagnosed hilar cholangiocarcinoma, which is T1N0M0 according to the Union for International Cancer Control (UICC) classification. Extended left lobectomy with hepaticojejunostomy was performed. The tumour was pathologically diagnosed with biliary intraepithelial neoplasia at the hilar left part of the bile duct (Fig. 2).

## 3 Postoperative course

**Fig. 3:**
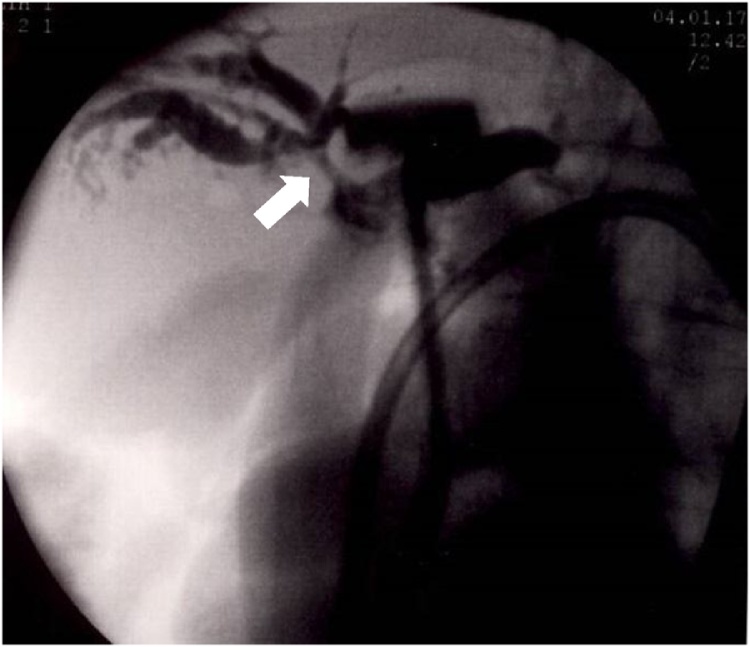
Abbreviation; PTCD: percutaneous trans-hepatic cholangiodrainage. This was the cholangiography by injection via PTCD tube. There was a narrow portion at the hepaticojejunostomy as the white arrow head pointed.

**Fig. 4:**
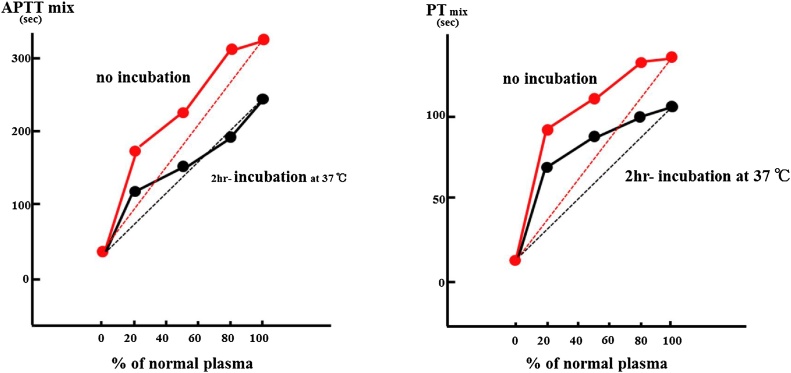
At 40 POD, the cross-mixing test was carried out to detect the causes of significant decreases of all coagulation factors. APTT tests are performed on series of mixtures of patient plasma and normal plasma. The result demonstrated there is a convex upward in an immediate reaction as shown in no incubation. The pattern was also shown in delayed reaction as 2hr-incubation at 37 °. Similarly, as for PT test, there is a convex upward in an immediate reaction as no incubation. In addition, the line pattern of delayed reaction illustrated a considerable convex upward pattern. These visualised results suggested the existence of coagulation inhibitors, rather than a deficiency of coagulation factors, since the incubation with the normal plasma significantly delayed the recovery of coagulation time.

**Fig. 5:**
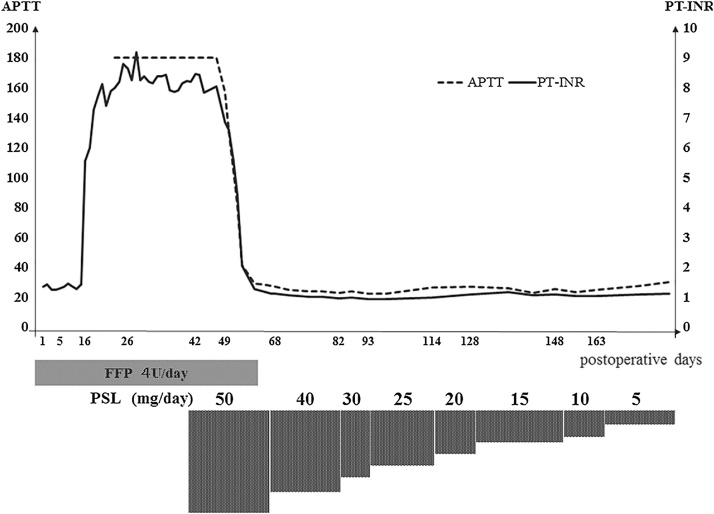
Abbreviations: POD: post-operative days, PSL: prednisolone, APTT: activated partial thromboplastin time, PT-INR: prothrombin time-international normalized ratio. We used average 4 units of fresh frozen plasma by 60POD. We started to use PSL from 42POD. The initial dose of PSL was 50 mg/day between 42POD and68POD, and then the figure was decreased to 40 mg/day from 68 to 82POD. This was followed by a decrease of 30 mg/day from 82POD to 93POD. The figure went down to 5 mg/day by 163POD. Due to using PSL, the level of coagulation factors, which were APTT and PT and then the figure was decreased to 40 mg/day from 68 to 82POD. This was followed by a decrease of 30 mg/day from 82POD to 93POD. The figure went down to 5 mg/day by 163POD. Due to using PSL, the level of coagulation factors, which were APTT and PT-INR, considerably decreased and settled between 29POD and 68POD.

The postoperative course was uneventful, and the patient was discharged at 19 post-operative days (POD). However, beginning at 35POD, the patient suffered from a high fever and was admitted to our hospital at 41POD. We determined that the reason was severe repetitive cholangitis because of a slight narrowing at the hepaticojejunostomy (Fig. 3). After several examinations and transhepatic cholangiodrainage (PTCD), the patient’s fever resolved. At 85POD, the narrow part was removed and reconstructed. The fever recurred by 6POD, and *Enterobacter cloacae* was detected in the abdominal drain. Levofloxacin was administered. In addition, surgical site infection was confirmed at 9POD. The high fever resolved by 16POD. Meanwhile, the level of PT% suddenly went down to 7%, and the figure marginally decreased to under 5% by 18POD (Table 1). We suspected there was an initial change in the extent of liver failure, and fresh frozen plasma (FFP) as well as vitamin K had been administered during this time. In spite of these treatments, the patient displayed repetitive bleeding; namely, a severe nosebleed at 24POD, melena at 26POD and a massive wound bleed at 31POD. Although AST, ALT and ALP had been almost normal during the days the patient displayed sickness, the levels of his all coagulation factors went down significantly (Table 2). We used an average of four units of FFP as a compensating therapy. Because the levels of coagulation factors did not recover, we tried to determine the cause of these considerable drops. On 40POD, a cross-mixing test was carried out, and we obtained visualised results. The line patterns suggested the existence of coagulation inhibitors rather than a deficiency in the coagulation factors. This was because the incubation with normal plasma significantly delayed coagulation time (Fig. 4). Based on these results, we finally identified the inhibition of all coagulation factors after the surgery as a cause of the patient’s symptoms. Based on treatments of autoimmune diseases, we prescribed prednisolone (PSL) beginning at 42POD. The initial dose was 50 mg/day between 42POD and 68POD, which was then decreased to 40 mg/day from 68POD to 82POD. This was followed by a dose reduction to 30 mg/day by 93POD and gradual decreases to 5 mg/day by 163POD (Fig. 5). Using PSL, the levels of coagulation factors went up beginning at 60POD. Since then, there have not been any complications with respect to bleeding. PSL has been used at a dose of 5 mg/day for about 2 years.

**Table 2:** The level of each coagulation factor.

Variable (coagulation factor)	Value	Normal range (%)
II	**<3**	75–135
V	**<3**	70–135
VII	**20**	75–140
VIII	**<1**	60–150
IX	**3**	70–130
X	**12**	70–130
XI	**3**	75–145
XII	**6**	50–150
vWf	**264**	60k170
Anti-nuclear antibody	20	<20

Abbreviation: vWf: von Willebrand factor.

Each coagulation factor was described with the normal range. There were significantly lower than the normal figures.

## 4 Discussion

We experienced a massive bleed in a patient with a deficiency in all coagulation factors. To our knowledge, this is the first report of acquired inhibitors for all coagulation factors associated with surgery.

In general, when clinicians encounter a patient with massive bleeding during the post-operative course, they may suspect that the possible causes are infection, initial stages of liver failure or something related to the surgical techniques. We initially doubted that there were problems in the patient’s liver function because of a significant decrease in the level of PT%, which is routinely examined in order to assess liver function [7]. We had examined liver function repeatedly over the course of the patient’s recovery, and the patient continued to spend days suffering from repetitive bleeding. If we had diagnosed the inhibition of all coagulation factors early on, we would have provided a better outcome using PSL and would not have had to use a large amount of FFP.

The present case presented with severe decreases in all coagulation factors during the post-operative course. We believe the major reason for this was the influence of the pre-malignant tumour itself and damage from surgery. In support of this, some previous studies have shown that inhibitors of coagulation factors are present in patients suffering from a malignant tumour, infection and physical damage [8,9]. Most reports are related to acquired haemophilia A caused by a deficiency in factor VIII (8), whereas only two previous cases were associated with the inhibition of multiple coagulation factors. One such case occurred in HCC [10] and another occurred in a patient with oral cancer [11]. Nevertheless, there is no literature showing acquired inhibition of all coagulation factors.

With regard to disease mechanism, there is a report describing the imbalance of coagulation factors as a result of tumour progression [12]. The authors showed the production and secretion of pro-coagulant factors affected by tumor biology and cancer-associated thrombosis, and ectopic thrombin production also affected tumour progression by influencing proliferation rate, angiogenesis, invasion and metastasis. From this point of view, we hypothesized that the pre-malignant tumour and surgical damage might inhibit the coagulation factors and lead to a reversible autoimmune reaction as described in these previous case reports.

As shown in our case, due to the rarity of acquired inhibitors of coagulation factors, the diagnosis is often difficult and delayed. Nonetheless, a prompt diagnosis of specific inhibitors is mandatory for starting an appropriate treatment aimed at overcoming the deficient factor [13]. In the present case, after several examinations, we finally obtained an accurate diagnosis by using a cross-mixing test. This test has been used in order to elucidate the causes of prolongation of PT as well as APTT since 2008 [14]. Recently, it has become a vital test for the diagnosis of lupus anticoagulants [15]. The examination identifies three potential causes: the existence of antiphospholipid antibodies, coagulation factor deficiencies or the existence of inhibitors. APTT tests can be performed on a series of mixtures of patient plasma and normal plasma. The procedure involving a cross-mixing test and normal plasma at various ratios with the results are visualized as a graph by connecting the APTT values. Thanks to the examination, we finally obtained an accurate diagnosis (Fig. 4).

Previous studies identified acquired vitamin K-dependent coagulation factor deficiency as a cause of bleeding in patients, and they indicated steroid pulse therapy as a treatment [[16], [17], [18]]. In addition, immunosuppressive drugs have a positive effect on acquired inhibitors of coagulation factors, which were associated with gastroenterological cancer [[19], [20], [21], [22], [23], [24]], and there are also a number of reports describing the benefits of steroid pulse therapy for specific autoimmune diseases [[25], [26], [27], [28]]. Admittedly, we hesitated to undertake the treatment because this patient had a high fever with a suspicious infection after the second surgery. However, we also struggled to manage a massive bleed with a considerable decrease in anticoagulant factors at the time. For this latter reason, we decided to use 50 mg PSL per day as the initial dose, which successfully increased of the levels of anticoagulant factors.

## 5 Conclusion

We have described the first case of acquired inhibition of all coagulation factors associated with extended left lobectomy. The reported treatment and examination will help clinicians explore additional reasons for massive bleeding after a severe physical injury.

## Conflicts of interest

I have no financial relationships to disclose and there are not any financial and personal relationships with other people or organisations about all authors.

## Sources of funding

I have no financial relationships to disclose and there is not any sponsor with funding.

## Ethical approval

Our reported case here involves a sufficient ethnical level. The approval has been given by the ethics committee in our institution as the assigned reference number 1810-001.

## Consent

Fully informed consent was obtained with some document. Written informed consent was obtained from the patient for publication of this case report and accompanying images. A copy of the written consent is available for review by the Editor-in-Chief of this journal on request.

## Author contribution

Yuhki Sakuraoka MD, PhD: Literature review and writing the article. Keiichi Kubota MD, PhD: Editing the article and he per-formed the surgery. All authors significantly contributed to revising the manuscript. They have read and approved this manuscript.

## Registration of research studies

Our reported case here is not research study. This is a case report. However, the approval has been given by the ethics committee in our institution as the assigned reference number 1810-001.

## Guarantor

Dr Yuhki Sakuraoka is the Guarantor of this report and has full responsibility to it.

## Provenance and peer review

Not commissioned, externally peer-reviewed
